# [Corrigendum] EGFR and ERK activation resists flavonoid quercetin-induced anticancer activities in human cervical cancer cells *in vitro*

**DOI:** 10.3892/ol.2026.15642

**Published:** 2026-05-08

**Authors:** Xin Chen, Pengli Xu, Huijun Zhang, Xiaosan Su, Lihua Guo, Xuhong Zhou, Junliang Wang, Peng Huang, Qingzhi Zhang, Ruifen Sun

Oncol Lett 22: 754, 2021; DOI: 10.3892/ol.2021.13015

Subsequently to the publication of the above paper, an interested reader drew to the authors’ attention that, concerning the cell invasion assay data shown in Fig. 4B on p. 7, the ‘0 h’ panels for the HeLa and SiHa cell lines showed a large overlapping section of data, such that these data appeared to have been derived from the same original source where the results from the two different cell lines were intended to have been portrayed. Additionally, upon performing an independent analysis of the data in this paper in the Editorial Office, it came to light that the Tubulin western bands in the ‘40’ and ‘80’-labelled lanes of Fig. 5C (for the HeLa cell line experiments) were strikingly similar to the Tubulin bands in the ‘0’ and ‘20’-labelled lanes in Fig. 3E (also for the HeLa cell line experiments).

The authors were able to re-examine their original data, and realized that the data included in Figs. 4B and 5C were inadvertently selected incorrectly. The revised versions of Figs. 4 and 5, now showing the correct data for the ‘0 h’ invasion assay data panel for the SiHa cell line and the Tubulin western blots for the HeLa cell line in Fig. 5C, are shown on the next two pages. The authors regret the errors that were made while compiling the original figures, and are grateful to the editor of *Oncology Letters* for allowing them the opportunity to publish this Corrigendum. Note that the errors in this pair of figures did not have a significant impact on the conclusions reached in this study. All the authors agree with the publication of this corrigendum; furthermore, they apologize to the readership for any inconvenience caused.

## Figures and Tables

**Figure 4.**
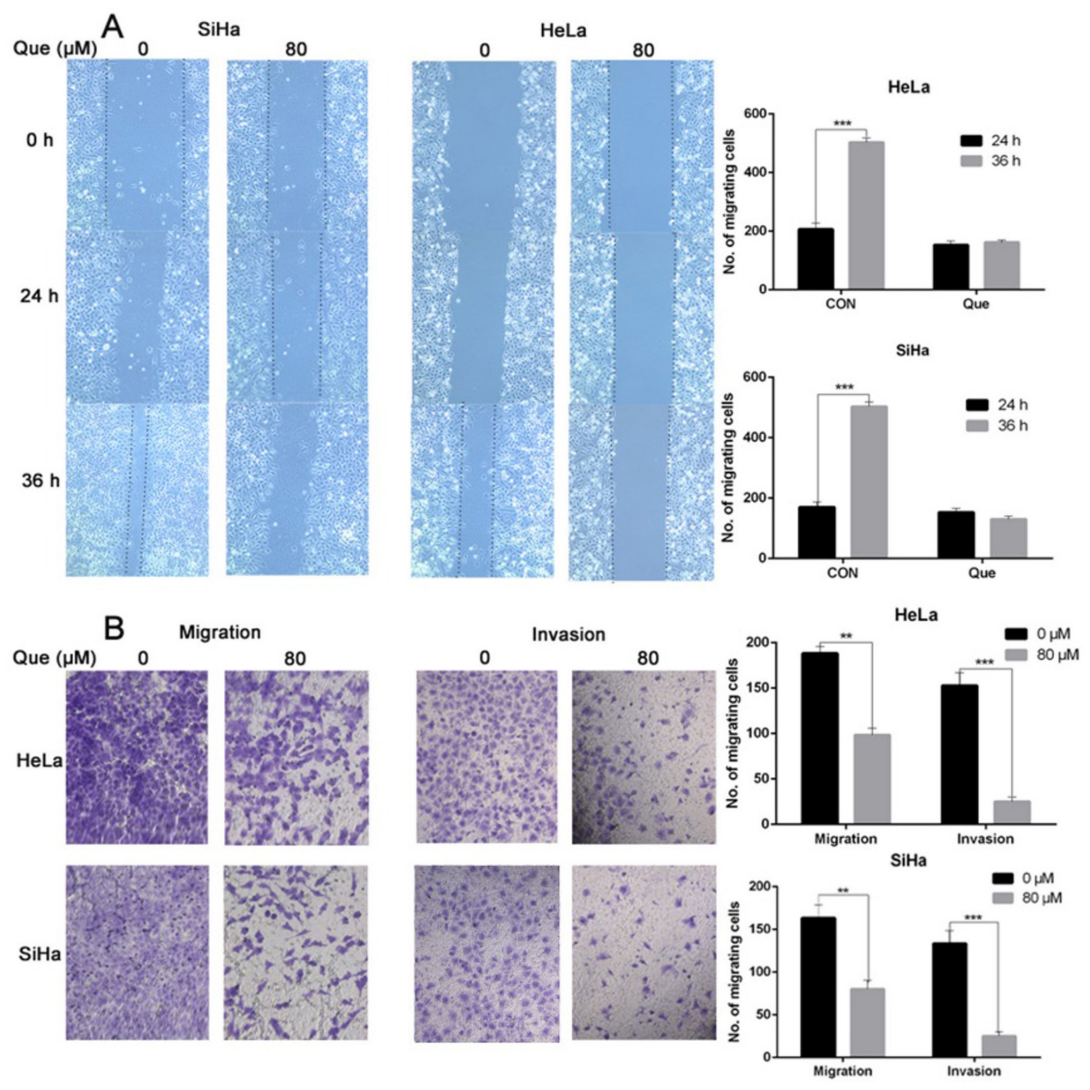
Que inhibits the migration and invasion of the cervical cancer cell lines. (A) The cells were treated with 0 or 80 µM Que for 0, 24 and 36 h, then the degree of wound healing was measured. (B) In total, ~10,000 SiHa and HeLa cells were seeded in the Transwell chamber in 200 µl FBS-free medium with the indicated concentration of Que for 48 h. Matrigel was added onto the microporous membrane in advance to simulate the environment of the extracellular matrix was only used for the invasion assay, and then the cells that penetrated the membrane were stained with crystal violet for subsequent examination. n=3. **P<0.01, ***P<0.001. Que, quercetin.

**Figure 5.**
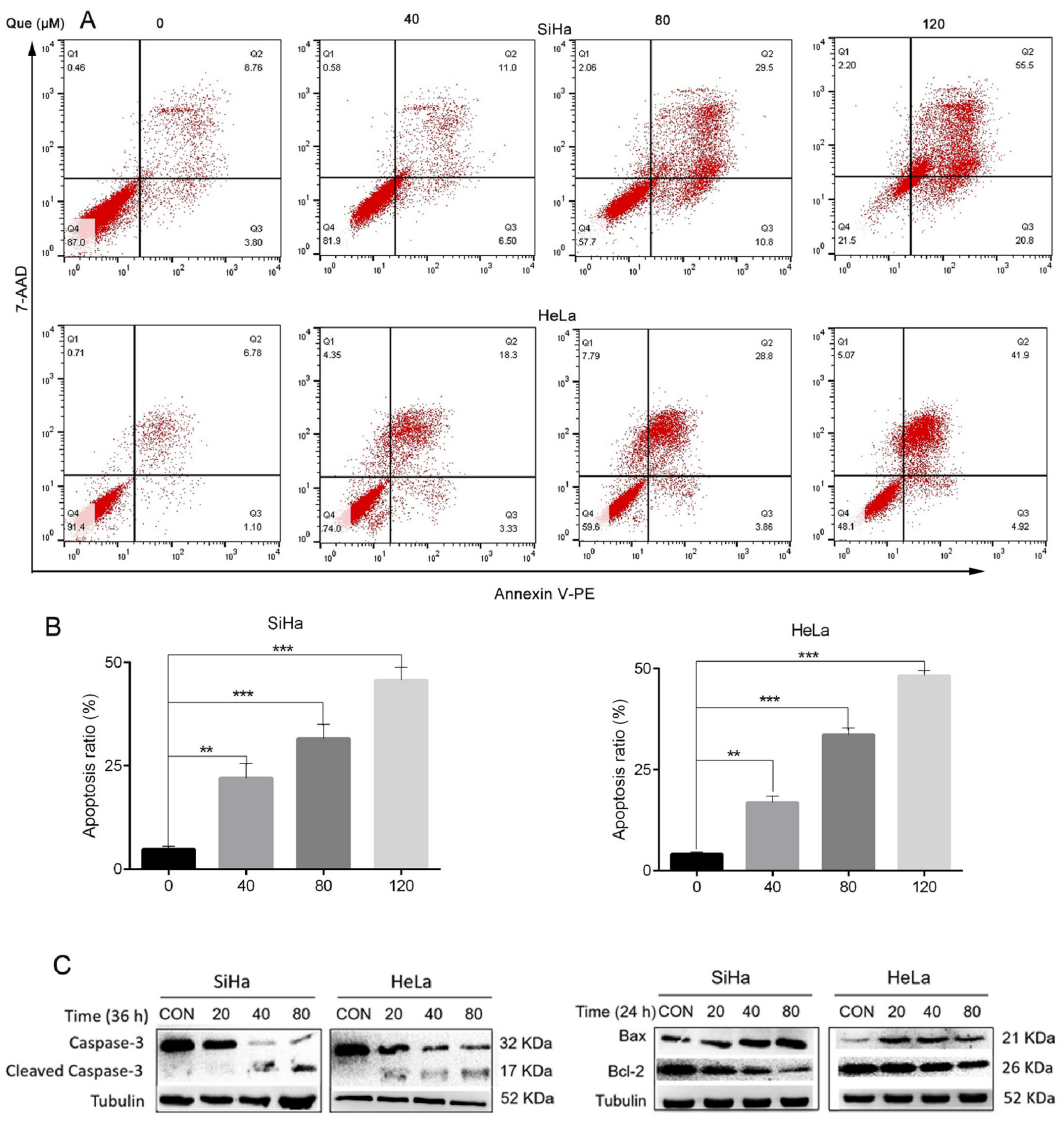
Que promotes cervical cancer cell apoptosis and affects the expression levels of apoptosis-related proteins. (A) The cervical cancer cells were treated with Que (0, 40, 80 and 120 µM) for 24 h, then flow cytometry analysis was performed to investigate cell apoptosis and the difference was (B) statistically analyzed. (C) The cervical cancer cell lines were treated with Que (0, 20, 40 and 80 µM) for 36 h, then the protein expression levels were examined using western blot analysis. Tubulin was used as the loading control. n=3. **P<0.01, ***P<0.001. Que, quercetin.

